# Impaired phrenic nerve axon development and diaphragm neuromuscular junction formation in embryonic *Cyfip2*-null mice

**DOI:** 10.1186/s13041-026-01301-6

**Published:** 2026-04-26

**Authors:** Su Yeon Kim, Jun Young Oh, U Suk Kim, Ruiying Ma, Yoonhee Kim, Kihoon Han

**Affiliations:** 1 Department of Neuroscience, Korea University College of Medicine, 73, Goryeodae-ro, Seongbuk-gu, Seoul, 02841 Republic of Korea; 2 Department of Biomedical Sciences, Korea University College of Medicine, Seoul, Republic of Korea

**Keywords:** CYFIP2, Phrenic nerve, Axon, Diaphragm, Neuromuscular junction, Embryo

## Abstract

Pathogenic variants in *CYFIP2* cause developmental and epileptic encephalopathy 65 (DEE65) and have been predominantly investigated in the context of central nervous system dysfunction. However, emerging clinical evidence suggests that peripheral nervous system (PNS) involvement may also contribute to disease manifestations. To explore this possibility, we examined the role of CYFIP2 in the development of the phrenic neuromuscular system, which is essential for neonatal respiration. Because conventional *Cyfip2*-null (*Cyfip2*^*−/−*^) mice exhibit perinatal lethality, we analyzed phrenic nerve axon development and diaphragm neuromuscular junction (NMJ) formation in embryonic mice. At embryonic day 16.5, *Cyfip2*^*−/−*^ embryos displayed significantly reduced phrenic nerve axon length and branching compared to wild-type controls. Postsynaptic acetylcholine receptor (AChR) clustering in *Cyfip2*^*−/−*^ diaphragms showed spatial heterogeneity: sparse regions exhibited a significant increase in endplate bandwidth, whereas dense regions showed a decreasing trend. Further analysis using synaptophysin and α-bungarotoxin labeling revealed reduced pre- and post-synaptic puncta density and decreased colocalization, despite preserved puncta intensity and volume, indicating impaired synaptic organization. Together, these findings demonstrate that CYFIP2 is required for proper phrenic nerve innervation and NMJ organization during embryonic development. This study extends the functional scope of CYFIP2 to the PNS and establishes the diaphragm as a tractable model for investigating peripheral mechanisms underlying *CYFIP2*-associated neurodevelopmental disorders.

**Supplementary Information:**

The online version contains supplementary material available at 10.1186/s13041-026-01301-6.

## Main text

Pathogenic variants in the Cytoplasmic FMR1-interacting protein 2 (*CYFIP2*) gene cause early-onset epileptic encephalopathy with developmental regression and are classified as developmental and epileptic encephalopathy 65 (DEE65) [1, 2]. At the molecular level, CYFIP2 is a core component of the Wiskott-Aldrich syndrome protein family verprolin-homologous protein (WAVE) regulatory complex (WRC), where it regulates neuronal actin cytoskeleton dynamics [3]. In addition, CYFIP2 interacts with multiple RNA-binding proteins, implicating it in RNA processing and translational control [4]. Consistent with these functions, studies using *Cyfip2* mouse models have largely focused on neuronal dysfunctions in the central nervous system [5–7]. In contrast, the roles of CYFIP2 in the development and function of the peripheral nervous system (PNS) remain poorly understood, despite clinical reports describing diverse PNS-related manifestations in individuals carrying pathogenic *CYFIP2* variants [8]. Addressing this gap is therefore important for a more complete understanding of *CYFIP2*-associated neurodevelopmental disorders.

Conventional *Cyfip2*-null (*Cyfip2*^*−/−*^) mice exhibit perinatal lethality [9]. Although the precise timing of death has not been directly determined, *Cyfip2*^*−/−*^ embryos are recovered at embryonic day 18.5 (E18.5) at near-Mendelian ratios, suggesting that lethality occurs shortly after birth. This pattern is consistent with impairment of physiological functions essential for postnatal survival, such as respiration. In this context, analysis of peripheral neural circuits critical for neonatal respiration provides a logical entry point to investigate CYFIP2 function in the peripheral nervous system. The phrenic nerve, which innervates the diaphragm, and the neuromuscular junctions (NMJs) formed within the diaphragm are indispensable for respiratory function [10]. Examination of phrenic nerve axon development and diaphragm NMJ formation therefore offers a focused framework to assess CYFIP2 function in the PNS. Consistent with this rationale, *Cyfip2* mRNA expression was detected in the embryonic mouse spinal cord, where the cell bodies of phrenic motor neurons reside, based on our previous in situ hybridization analysis [9] and interrogation of published single-cell RNA sequencing datasets (Additional file 1, Fig. S1a and b). Moreover, *Cyfip2* expression in the postnatal spinal cord was confirmed using data from the Allen Brain Atlas (Additional file 1, Fig. S1c).

**Fig. 1:**
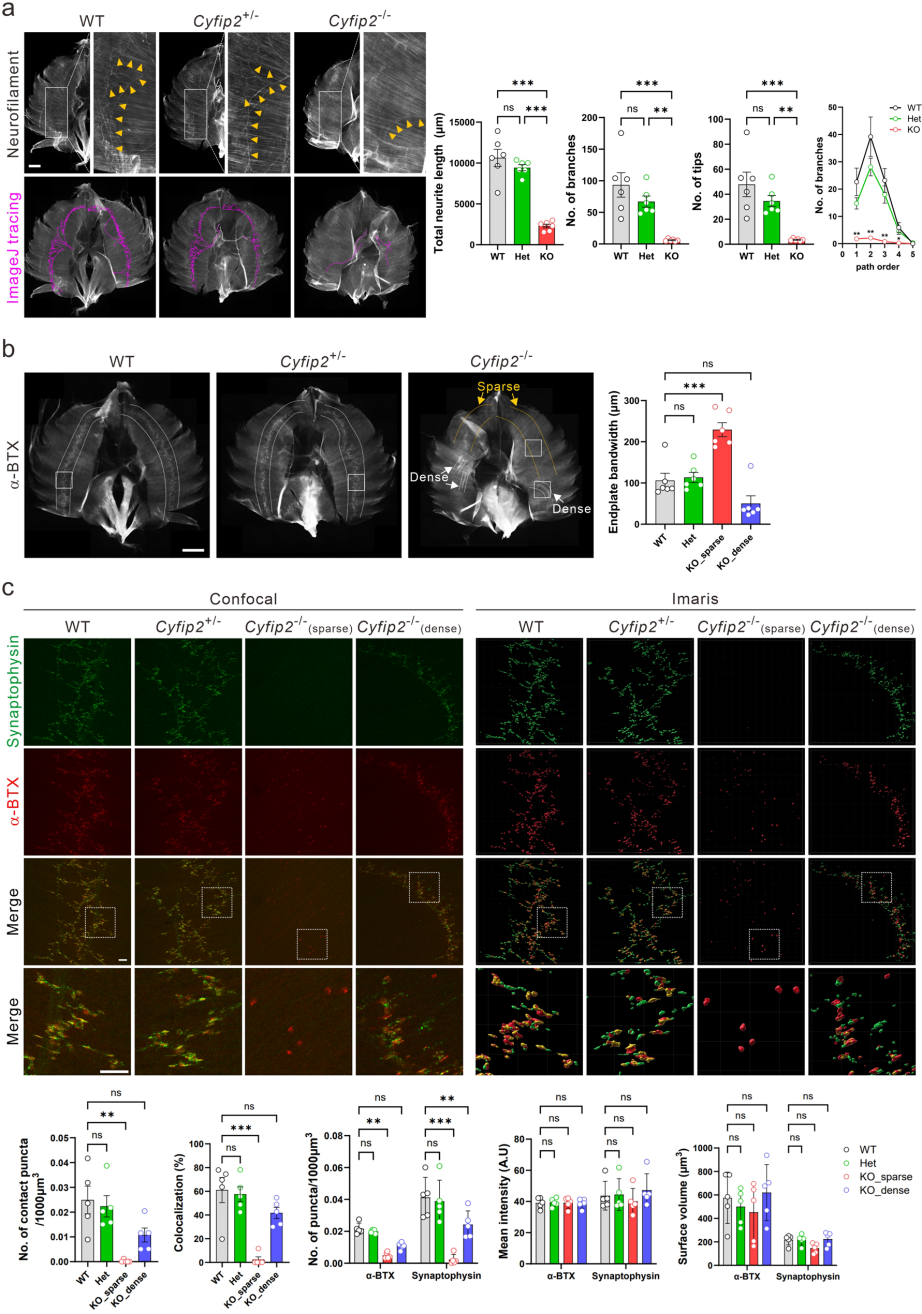
Loss of CYFIP2 impairs phrenic nerve innervation and neuromuscular junction organization in the diaphragm. **a** Representative neurofilament staining and traced images of whole-mount diaphragms from WT, *Cyfip2*^*+/−*^ (Het), and *Cyfip2*^*−/−*^ (KO) embryonic mice (E16.5), with corresponding quantification (*n* = 6 animals per genotype). Neurites are indicated with yellow arrowheads. Scale bar, 1 mm **b** Representative α-bungarotoxin (α-BTX) staining of whole-mount diaphragms from WT, *Cyfip2*^*+/−*^, and *Cyfip2*^*−/−*^ embryonic mice, with corresponding quantification of endplate bandwidth (*n* = 6 animals per genotype). The width of α-BTX-positive regions is indicated by white dotted lines. In *Cyfip2*^*−/−*^ samples, white and yellow dotted lines indicate densely and sparsely distributed endplates, respectively. White boxes denote regions shown in panel (c). Scaler bar, 1 mm. **c** Representative confocal images of pre- and post-synaptic signals at neuromuscular junctions, along with corresponding Imaris-rendered surfaces. Quantification of pre- and post-synaptic puncta parameters is shown (*n* = 5 animals per genotype). Scale bar, 40 μm. Data are presented as mean ± SEM. One-way ANOVA with Tukey post hoc test was used. **P < 0.01, ***P < 0.001; ns, not significant

Phrenic nerve axons innervating the diaphragm were visualized at E16.5 using anti-neurofilament immunostaining in wild-type (WT), *Cyfip2* heterozygous (*Cyfip2*^*+/−*^), and *Cyfip2*^*−/−*^ littermates (Fig. 1a). Quantitative analysis revealed significantly reduced axonal length and branching in *Cyfip2*^*−/−*^ mice compared to WT mice. *Cyfip2*^*+/−*^ mice showed a trend toward reduction; however, these changes did not reach statistical significance. Labeling of postsynaptic acetylcholine receptor (AChR) clusters with α-bungarotoxin (α-BTX), followed by quantification of endplate bandwidth, revealed no difference between WT and *Cyfip2*^*+/−*^ mice (Fig. 1b). In *Cyfip2*^*−/−*^ diaphragms, two distinct patterns of endplate distribution were observed. Endplate bandwidth was significantly increased in sparse regions, indicating a broader distribution of AChR clusters, whereas it showed a trend toward reduction in dense regions compared with WT mice. To further assess synaptic organization, synaptophysin immunostaining was performed together with α-BTX, and puncta parameters were quantified (Fig. 1c). No significant differences were detected between WT and *Cyfip2*^*+/−*^ mice in any of the measured parameters, including puncta density (number of puncta per unit volume), intensity (average intensity per punctum), volume (average volume per punctum), and colocalization (overlap of pre- and postsynaptic puncta). In contrast, *Cyfip2*^*−/−*^ mice showed no changes in puncta intensity or volume, but exhibited significant reductions in puncta density and colocalization. These reductions were more pronounced in regions with sparse endplate distribution compared to dense regions (Fig. 1c). To further investigate the differences between sparse and dense regions in *Cyfip2*^*−/−*^ mice, we examined triple staining (neurofilament, synaptophysin, and α-BTX) and found that neurofilament signals were largely absent in sparse regions (Additional file 1, Fig. S2). These observations suggest that AChR clusters in sparse regions may initially form through muscle-intrinsic mechanisms [11], but fail to undergo subsequent maturation due to the lack of axonal innervation. Collectively, these results demonstrate that CYFIP2 is required for proper phrenic nerve innervation and NMJ organization during embryonic development.

At the molecular level, further studies are needed to define how CYFIP2 regulates phrenic nerve innervation and diaphragm NMJ organization. One plausible mechanism involves actin cytoskeleton regulation through the WRC, which may influence axonal growth cone dynamics during motor axon extension [12]. In addition, given the interaction of CYFIP2 with multiple RNA-binding proteins, loss of CYFIP2 may affect local mRNA translation within developing axons, a process essential for axon growth and synapse formation [13, 14]. These cytoskeletal and translational mechanisms may act in concert to control axon development and NMJ organization. In parallel, it also remains possible that alterations in the number or spatial distribution of phrenic motor neurons may contribute to the phenotypes observed in this study. Addressing this possibility will require further analyses of the spinal cord, which will be an important direction for future investigation. Importantly, *CYFIP2* pathogenic variants may exert gain-of-function or dominant effects rather than simple loss-of-function [2, 15], warranting detailed mechanistic analyses. In this context, the diaphragm provides a tractable model to dissect CYFIP2 function in the PNS and may offer insight into the broader neurobiological basis of clinical manifestations.

## Supplementary Information

Below is the link to the electronic supplementary material.

Supplementary Material 1.

## Data Availability

The datasets used and analyzed in the current study are available from the corresponding author on reasonable request.

## Abbreviations

CYFIP2: Cytoplasmic FMR1-interacting protein 2
DEE65: Developmental and epileptic encephalopathy 65
E18.5: Embryonic day 18.5
NMJ: Neuromuscular junction
PNS: Peripheral nervous system
WAVE: Wiskott-Aldrich syndrome protein family verprolin-homologous protein
WRC: WAVE regulatory complex
WT: Wild-type
