# Supplementary material for: Impaired phrenic nerve axon development and diaphragm neuromuscular junction formation in embryonic Cyfip2-null mice
Source: Mol Brain. 2026 Apr 26;19:32. doi: 10.1186/s13041-026-01301-6 (PMC13130780; doi:10.1186/s13041-026-01301-6)
Supplement: Supplementary file 1 — Supplementary Material 1. [file 13041_2026_1301_MOESM1_ESM.docx]

**Additional File 1.**

**Impaired phrenic nerve axon development and diaphragm neuromuscular junction formation in embryonic *Cyfip2*-null mice**

Su Yeon Kim, Jun Young Oh, U Suk Kim, Ruiying Ma, Yoonhee Kim, Kihoon Han

**Supplementary Figures**


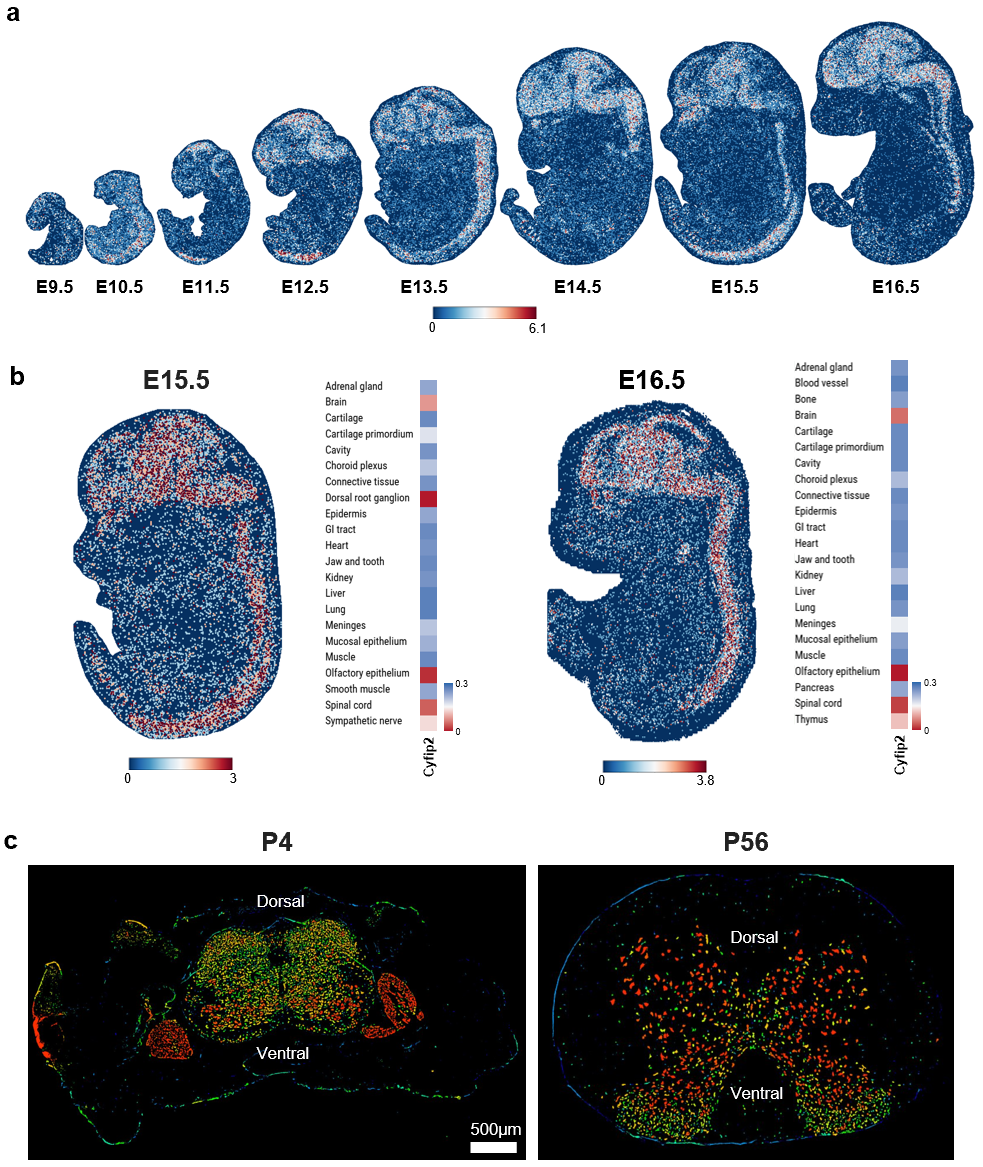


**Fig. S1. Expression pattern of *Cyfip2* mRNA in mouse embryos and spinal cord.**

(a) Spatial expression of *Cyfip2* across mouse embryonic stages. (b) Spatial visualization and corresponding heatmaps showing *Cyfip2* expression in embryonic day (E)15.5 and E16.5 embryos. Color scales represent log-normalized expression values. For heatmaps, colors indicate mean log-normalized expression levels. Blue denotes low expression and red denotes high expression. Data in (a) and (b) were obtained from the MOSTA database (<https://db.cngb.org/stomics/mosta/>) (1) and color schemes were modified for visualization. (c) In situ hybridization images demonstrating the expression of *Cyfip2* in the mouse spinal cord at postnatal day (P)4 and P56. Data were obtained from Allen Brain Atlas. Scale bar, 500 μm.


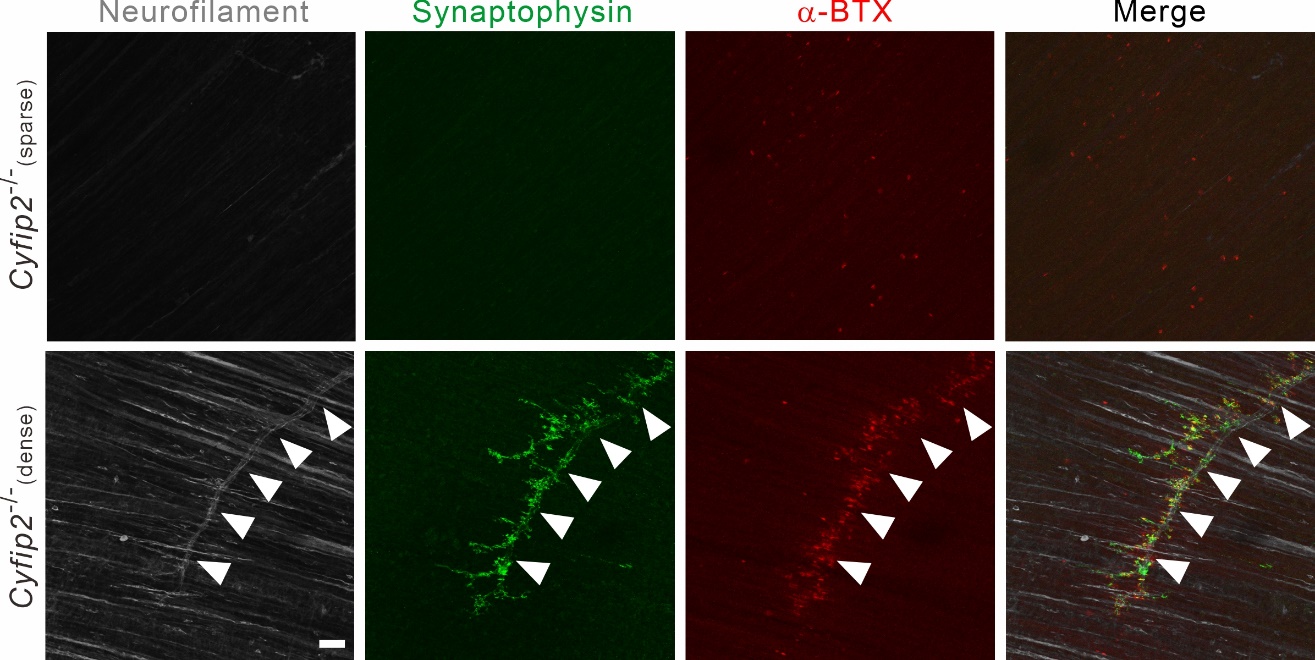


**Fig. S2. Lack of axonal innervation in the sparse region of *Cyfip2^−/−^* diaphragms.**

Representative confocal images of diaphragm from *Cyfip2^−/−^* embryos stained for neurofilament, synaptophysin, and α-bungarotoxin (α-BTX). Sparse region shows markedly reduced or absent neurofilament and synaptophysin signals, indicating a lack of axonal innervation, despite the presence of α-BTX-labeled AChR clusters. Scale bar, 40 μm.

**Materials and Methods**

**Animals**

The *Cyfip2* mouse line (*Cyfip2^tm1(KOMP)Vlcg^*) used in this study has been described previously (2-4). All mice were maintained on a C57BL/6N background. Wild-type, *Cyfip2* heterozygous, and *Cyfip2*-null littermates were generated by heterozygous intercrosses (*Cyfip2^+/-^* × *Cyfip2^+/−^*). All animal procedures were conducted in accordance with the research guidelines of the Korea University College of Medicine.

**Fluorescence immunohistochemistry**

For whole mount immunostaining of diaphragms, embryos were fixed in 4% paraformaldehyde (PFA) at 4°C overnight, and diaphragms were isolated as previously described (5). After phosphate-buffered saline (PBS) washes, the tissues were incubated in 0.1M glycine for 1h at room temperature (RT) and blocked in blocking buffer (2.5% BSA and 0.05% triton X-100 in PBS) for 1h at RT. Samples were incubated with anti-Neruofilament (1:500, DSHB, 2H3) and anti-Synaptophysin (1:300, Invitrogen, PA1-1043) antibodies at 4°C overnight, followed by incubation with AlexaFluor 488- or 555-conjuagted secondary antibodies (1:500, Invitrogen) and α-bungarotoxin (2 μg/1 ml, biotium, 00004) in blocking buffer for 4h at RT. Finally, samples were mounted for imaging.

**Image acquisition and image quantification**

Whole diaphragm images were obtained with a Zeiss AxioScanZ1 digital slide scanner equipped with a Plan Apochromat 10x (0.45 NA) lens. Individual neurites were manually traced and analyzed using Simple Neurite Tracer (SNT) (6) plugin in ImageJ. Branching analysis was performed up to fourth-order branches. Given that anti-neurofilament antibody staining produced partial background labeling of diaphragm muscle tissue, axon tracing was performed with particular caution. For axon tracing, analysis was initiated from the thickest and most clearly distinguishable axon fibers located in the central region of the diaphragm. Upon higher magnification, these axons were reliably distinguished from muscle fibers, which exhibit uniformly parallel alignment; accordingly, linear structures aligned with the direction of muscle fibers were excluded from quantification in all analyzed images. To minimize potential bias, axon tracing and quantification were performed in a blinded manner by an investigator who was not involved in the staining procedure and was unaware of the genotype. To quantify the distribution of acetylcholine receptor (AChR) clusters in the diaphragm, endplate bandwidth (EPBW) was analyzed using ImageJ software. Briefly, line profiles were measured across the diaphragm to obtain the fluorescence intensity of the α-bungarotoxin signal. The boundaries of the endplate band were defined by a threshold of 60% of the maximum fluorescence intensity within the profile. The EPBW was determined by calculating the distance between the first and last points where the intensity exceeded the predefined threshold. To assess pre- and post-synaptic colocalization, the same samples were imaged on a Zeiss LSM900 confocal microscope equipped with a 10x (0.45 NA) objective. Pre- and Post-synaptic puncta were quantified using the Surface tool in Imaris. Colocalization was defined as an overlapping volume ration greater than 0.5 between pre- and post-synaptic surfaces.

**Statistics**

All statistical analysis was performed using the latest version of GraphPad Prism and graphs were drawn with the same software. All analysis was performed on raw imaging data without any adjustments. Images in figures have been adjusted for brightness and contrast, identical for all groups compared.

**References**

1. Chen A, Liao S, Cheng M, Ma K, Wu L, Lai Y, et al. Spatiotemporal transcriptomic atlas of mouse organogenesis using DNA nanoball-patterned arrays. Cell. 2022;185(10):1777-92 e21.

2. Han K, Chen H, Gennarino VA, Richman R, Lu HC, Zoghbi HY. Fragile X-like behaviors and abnormal cortical dendritic spines in cytoplasmic FMR1-interacting protein 2-mutant mice. Hum Mol Genet. 2015;24(7):1813-23.

3. Lee SH, Zhang Y, Park J, Kim B, Kim Y, Lee SH, et al. Haploinsufficiency of Cyfip2 Causes Lithium-Responsive Prefrontal Dysfunction. Ann Neurol. 2020;88(3):526-43.

4. Zhang Y, Kang H, Lee Y, Kim Y, Lee B, Kim JY, et al. Smaller Body Size, Early Postnatal Lethality, and Cortical Extracellular Matrix-Related Gene Expression Changes of Cyfip2-Null Embryonic Mice. Front Mol Neurosci. 2018;11:482.

5. Sha R, Wang ZZ, You X, Liu YG, Xie ZQ, Feng Y. Whole-mount Staining of Mouse Diaphragm Neuromuscular Junctions. Bio-Protocol. 2021;11(21).

6. Arshadi C, Gunther U, Eddison M, Harrington KIS, Ferreira TA. SNT: a unifying toolbox for quantification of neuronal anatomy. Nat Methods. 2021;18(4):374-7.
